# Sample Selection for Training Cascade Detectors

**DOI:** 10.1371/journal.pone.0133059

**Published:** 2015-07-21

**Authors:** Noelia Vállez, Oscar Deniz, Gloria Bueno

**Affiliations:** VISILAB Group, ETSI Industriales, University of Castilla—La Mancha, Ciudad Real, Spain; Xiamen University, CHINA

## Abstract

Automatic detection systems usually require large and representative training datasets in order to obtain good detection and false positive rates. Training datasets are such that the positive set has few samples and/or the negative set should represent anything except the object of interest. In this respect, the negative set typically contains orders of magnitude more images than the positive set. However, imbalanced training databases lead to biased classifiers. In this paper, we focus our attention on a negative sample selection method to properly balance the training data for cascade detectors. The method is based on the selection of the most informative false positive samples generated in one stage to feed the next stage. The results show that the proposed cascade detector with sample selection obtains on average better partial AUC and smaller standard deviation than the other compared cascade detectors.

## Introduction

Viola and Jones proposed an efficient cascade framework that rapidly discards negatives and spends more time in positive candidates. The cascade framework is one of the most successful practical products of vision research [1]. Some authors have proposed modifications to the original cascade detector in order to improve the detection rate while maintaining or reducing the false positive rate (see next Section). However, when the training dataset is imbalanced (the number of negative samples far outnumbers the positive ones) classifier performance is reduced [2]. In some detection problems such as face detection, medical lesion detection, or pedestrian detection the negative set typically contains orders of magnitude more images than the positive set. These training datasets are such that the positive set has too few samples and/or the negative set should represent anything except the object of interest and this can give rise to biased classifiers. Other authors have been demonstrated that negative sample selection improves classification results [3].

Classifiers trained with imbalanced datasets can have a good error rate on the majority class but not as good in the other. This is due to the classifiers training process which attempts to obtain a good global error rate in most cases [4]. Therefore, a number of samples from the majority class (the negative class) must be selected in order to obtain well-balanced training datasets.

In order to select samples, the most common strategy in practice is random selection. However, random selection may lead to a non-representative dataset [5]. Therefore, specific selection strategies have been proposed in the literature for discarding redundant information [6] or improving classification results [7]. Moreover, to deal with imbalanced datasets, another option is to obtain more samples of the minority class through oversampling [7].

In contrast to emerging classification algorithms such as SRC (Sparse Representation Classifier) or CNN (Convolutional Neural Networks) [8, 9], cascade detectors are currently widely used in real time detection and classification problems. However, cascade detector solutions rely on the assumption of the independence between stages which is not held in practice [10]. Therefore, this paper focus on improving the cascade detector framework. A cascade detector is proposed in which a selection is made of the false positives generated by the previous stage, from the pool of all false positives generated. The subset of false positives is selected based on their associated confidence scores obtained in the previous stage. With this method, each stage uses the same number of negative samples than positive samples, thus keeping stage classifiers balanced while maximizing stage independence. The methodology has been tested with three different datasets for detecting faces, pedestrians and breast lesions on mammograms [4, 11, 12], and has been compared with Viola and Jones [1], Chen and Chen [13], and Soft [14] cascade detectors in order to check that this sample selection is better than using random samples to train new stages.

The rest of the paper is organized as follows. Section Background makes an overview of previous work focused on improving the original cascade framework. Section Materials and Methods describes the proposed cascade framework and the databases used. Finally, the results of the comparative and the main conclusions are described in the last two sections respectively.

## Background

**Fig 1:**
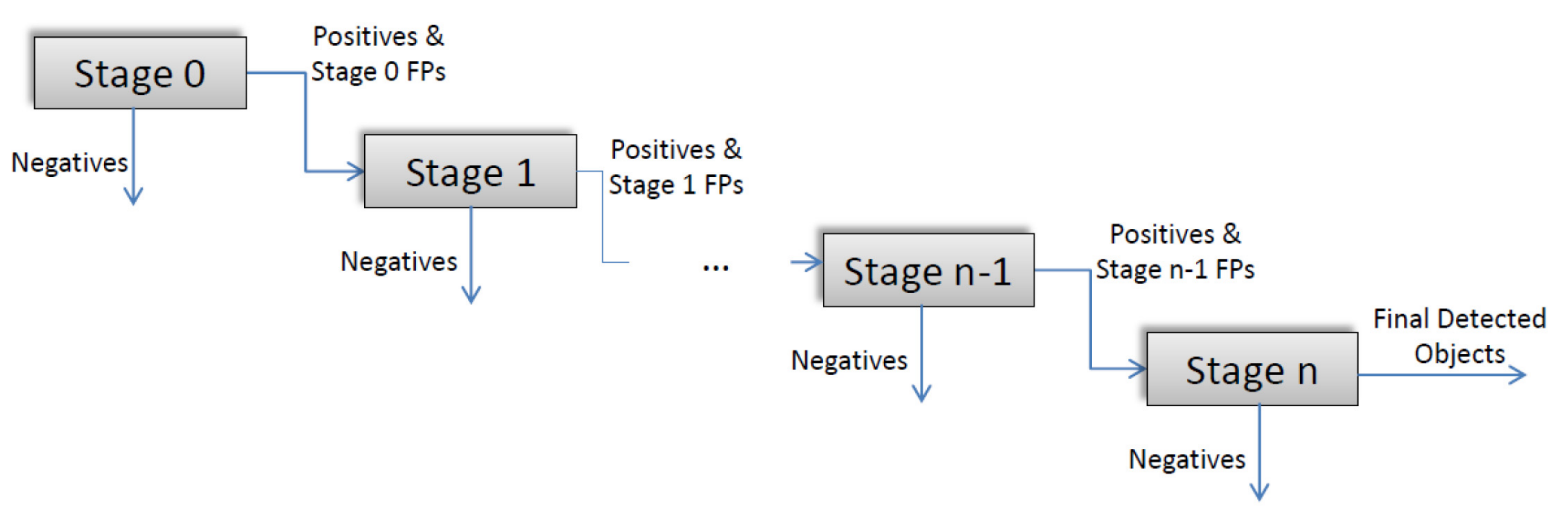
Viola-Jones cascade framework.

Cascade detectors were introduced to perform object detection efficiently. The cascade structure is a set of classifiers with increasing complexities (simple and fast classifiers are on the first stages). If a stage’s decision is positive the sample proceeds to the next stage. Otherwise it is discarded without further processing (Fig 1). Thus, not all the features and stages are computed and executed for all samples. In general, cascade detectors operate with high accuracyand are currently used for several types of detection problems [10].

The first cascade detector was proposed by Viola and Jones. It consists of a cascade of boosted classifiers based on Haar-like features which act as a single classifier [1]. In the original framework, AdaBoost (Adaptive Boosting) was selected as the boosting algorithm for building the stages [15–17]. By establishing the stage’s target minimum Dr close to 1 and the maximum FPr to 0.5, cascades rapidly eliminate easy images in the first few stages and maintain positive and difficult negative images until the last stages. Therefore, a cascade can be seen as a process that detects positive samples by continuously filtering false positives. During the detection step of the Viola-Jones cascade detector, a sliding-window is shifted over the input image and the Haar-like feature set of the actual detector stage is calculated. Thus, only the needed features are calculated each time.

Many works have extended the original cascade classifier to improve its effectiveness and efficiency. For example, Lienhart and Mydt in [18] proposed a cascade of Gentle AdaBoost stages where the modified training of the stage classifiers results in an increased training speed. However, training the new stages without considering any information from the previous ones could produce overfitting. Sochman and Matas [19] proposed inserting the previous stage classifier at the start of the new stage before training it. Thus, previous information is taken into account when building a new stage. Deng and Su [20] designed a cascade that improves the true positive rate while keeping the false alarm rate. If an image or a region is discarded at a given stage, in this case it is not labelled directly as negative but it is labelled as *probable positive*. These samples are given a positive-sample likelihood according to the number of stages that they have passed and the final classifier confidence assigned to them. Finally, a vote is performed to obtain the final labels of these probable positives. Chen and Chen proposed a method that can reduce the number of features needed at the cost of increasing the complexity of the calculations [13]. This cascade detector combines AdaBoost and SVM stages. The authors proposed adding some intermediate classifiers (called meta-stage classifiers) that use the inter-stage information to learn new classification boundaries and improve the results. Similarly to the Chen-Chen cascade detector, Cheng and Jhan combined AdaBoost and SVM Stages [21] by modifying the original Viola-Jones cascade and replacing AdaBoost stages by SVM stages when the number of features used is greater than a given threshold.

Other authors try to find a way to globally optimize cascades. Dundar and Bi [22] proposed a joint cascade training method in which the parameters for a stage classifier were updated depending on the performance of the other classifiers. In this case, a negative candidate sample is classified as negative by the cascade when it is discarded by almost one of the stages. On the contrary, to be labelled as positive, the sample is required to be accepted by all of them. Oliveira *et al*. [23] formulate the problem of finding the cascade thresholds as an optimization problem. To solve it, they used PSO (Particle Swarm Optimization) [24, 25]. In other works, such as the ones from Raykar and Krishnapuram [26], Pujara *et al*. [27], or Saberian [28] the cascade is globally optimized considering the trade-off between accuracy and cost and a function relating these two parameters is optimized.

Finally, other proposed methods are based on single-stage cascades that reduce computational time. However, this improvement does not always entail good detection and false positive rates [29]. Grossmann [30] proposed building a cascade classifier from a single classifier created by Boosting. The idea was to compute a subset of weak classifiers from a classifier with many features and test the samples with it. If the sample is positive another subset is chosen and tested. Following the same idea, in [14], Bourdev and Brandt proposed a one-stage cascade detector called Soft cascade. The idea in this case is to train a long AdaBoost stage using *T* weak learners.

## Materials and Methods

Since we focus our attention on creating a balanced classifier, the selection of some of the negative samples (the same as positive samples) to train additional stages is proposed.

### Proposed method

The selection of some samples from the negative set is not a trivial task [31]. While positive training images can be preserved over the new stages, previous stage generated true negatives should be discarded and false positives need to be selected so as to improve accuracy.

If a cascade is composed of several independent stages, the final detection rate, *D*, and the false positive rate, *F*, are given by the product of stage rates as follows [1]:

$$\begin{array}{c} D=\prod_{i=0}^{N}D_{i} \end{array}$$ (1)

$$\begin{array}{c} F=\prod_{i=0}^{N}F_{i} \end{array}$$ (2)

where *N* is the number of stages in the cascade.

Since a cascade composed of stages that are independent from each other achieves better results [1, 10], the assumption in this work is that a stage must be trained using the FPs that maximize the independence from the previous stage.

Let us now consider the following situation. A cascade has been trained up to a given stage, and our aim is to select some of the false positives at that point to train the next stage. Let *C* be the cascade, *H* the stage to be added and X the set of false positive samples selected for training *H*. *C* and *H* are conditionally independent given *X*, written as *C* ⊥ *H* ∣ *X*, if and only if:

$$\begin{array}{c} P(C\cap H|X)=P(C|X)P(H|X) \end{array}$$ (3)

Let us now consider the following two conditions:

a) *C* and *X* are independent events. Therefore,
  $$\begin{array}{c} P(C\cap X)=P(C|X)P(X)=P(C)P(X) \end{array}$$ (4)
  and
  $$\begin{array}{c} P(C|X)=P(C) \end{array}$$ (5)
b) *H* and *X* are dependent events. Thus, the conditional probability of *H* given *X* is:
  $$\begin{array}{c} P(H|X)=1, \end{array}$$ (6)
  their joint probability is defined as:
  $$\begin{array}{c} P(H\cap X)=P(H|X)P(X)=P(X) \end{array}$$ (7)
  and the intersection of these two events is:
  $$\begin{array}{c} H\cap X=X \end{array}$$ (8)

Since the joint probability of *C* and *H* given *X* can be defined based on the joint probability of *C*, *H*, and *X* and the probability of *X* as

$$\begin{array}{c} P(C\cap H|X)=\frac{P(C\cap H\cap X)}{P(X)} \end{array}$$ (9)

considering Eq (8), Eq (9) can be rewritten, in this case, as follows:

$$\begin{array}{c} P(C\cap H|X)=\frac{P(C\cap X)}{P(X)} \end{array}$$ (10)

At this point, considering Eq (4), *P* (*C* ∩ *H*|*X*) is equivalent to:

$$\begin{array}{c} P(C\cap H|X)=\frac{P(C)P(X)}{P(X)}=P(C) \end{array}$$ (11)

Finally, replacing Eqs (5) and (6) in Eq (11):

$$\begin{array}{c} P(C\cap H|X)=P(C)=P(C|X)\cdot 1=P(C|X)P(H|X) \end{array}$$ (12)

This demonstrates that conditions a) and b) above make Eq (3) an equality. In other words, the set of samples *X* that enforces conditions a) and b) make the new stage *H* independent.

With regards to condition a), the output of the classifier is the posterior probability *P* (*C* | *X*). When *C* and *X* are independent *P* (*C* ∩ *X*) = *P* (*C*) *P* (*X*) and, therefore *P* (*C* | *X*) = *P* (*C*). This means that the output of the classifier must not depend on the input samples. This occurs when the samples are on the classifier boundary, where the output of the classifier corresponds to random guessing. This means that condition a) can be imposed by means of the selection of the closest samples to the boundary of classifier *C*. On the other hand, condition b) is imposed by the training process itself due to the fact that the classifier to train is fed with the selected training samples.

**Fig 2:**
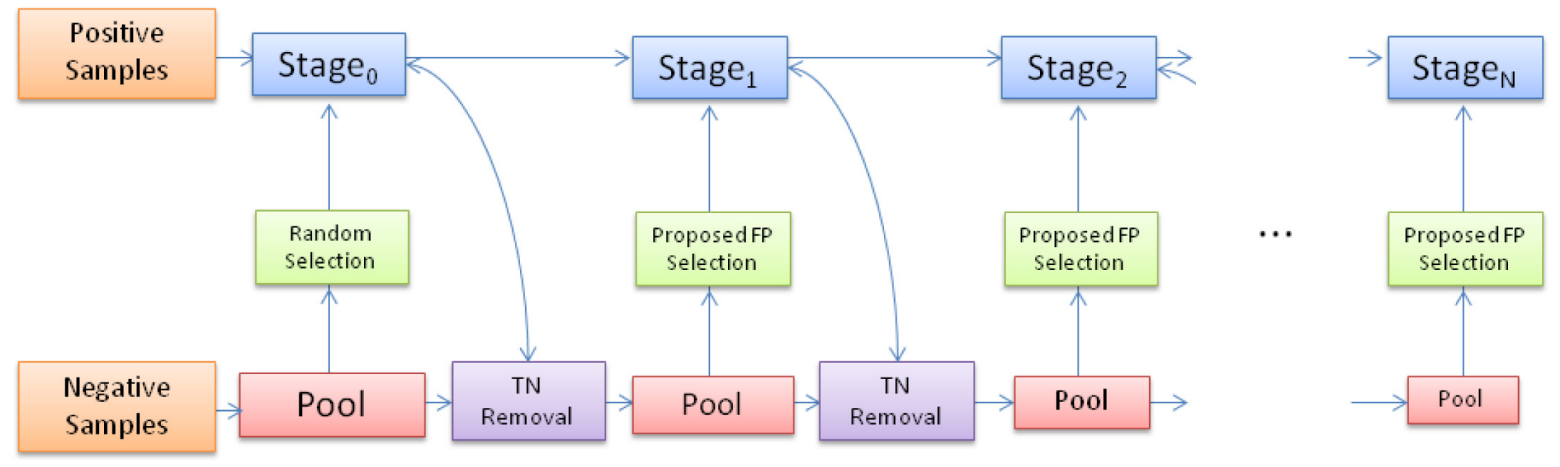
Proposed cascade framework training process.

Stages are trained using AdaBoost with decision stumps as weak learners. Fig 2 and Algorithm 1 in Table 1 show the proposed cascade with sample selection. After each cascade stage, some samples are selected from a pool of misclassified negative samples to create the stage training negative dataset. The selection is performed using the above criteria.

**Table 1:** Proposed cascade training algorithm.

**Require** *P* = positive dataset
*Pool* = negative dataset
*N* = random subset of negative dataset with the same size than *P*
*f* = maximum stage false positive rate
*d* = minimum stage detection rate
*F* = maximum global false positive rate
**Ensure** *H*(*x*) = final cascade classifier
*F*_0_ = 0
*D*_0_ = 0
*i* = 0
**while** *F*_*i*_ > *F* **do**
*i* = *i*+1
*n*_*i*_ = 0
*F*_*i*_ = *F*_*i*−1_
**while** *F*_*i*_ > f*F_*i*−1_ **do**
*n*_*i*_ = *n*_*i*_+1
*AdaBoost*(*P*, *N*, *n*_*i*_)
[*F*_*i*_, *D*_*i*_] ← *evaluate*()
**while** *D*_*i*_ < d*D_*i*−1_ **do**
*decrementClassifierThreshold*()
**end while**
**end while**
*newN* = {}
**if** *F*_*i*_ > *F* **then**
**for all** *Image *I* from *Pool** **do**
(*eval*, *confidence*) ← *evaluate*(*I*)
**if** *eval* = *Negative* **then**
*Pool*.*delete*(*I*)
**end if**
**end for**
**select** the size(*P*) samples **from** *Pool* with the smallest confidence values
**end if**
**end while**

### Image datasets

**Fig 3:**
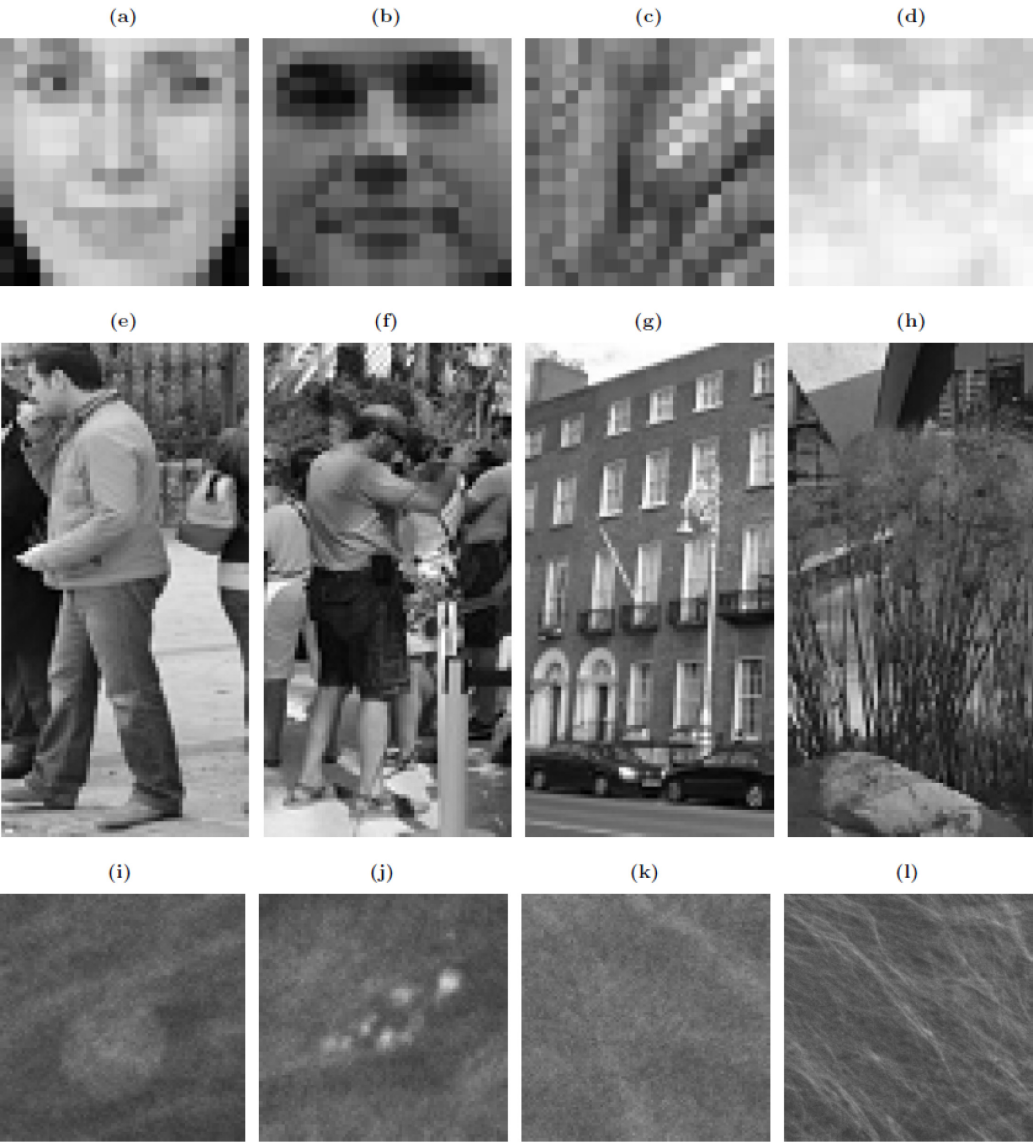
Image samples from used datasets. CBCL dataset similar images (a-d), INRIA dataset similar images (e-h), and images from mammography dataset (i-l). Images on the first two columns contain objects while the rest are negative samples.

To train and test the cascade detectors three different image datasets have been considered: the CBCL facial dataset [11], the INRIA dataset of pedestrians [12] and a mammography lesions dataset [4] (Fig 3).

The CBCL dataset is a public collection of images which includes a total of 31,022 images of which 2,901 contain faces and 28,121 contain different backgrounds. All images are in grey scale and have a resolution of 19 × 19 pixels.

The INRIA dataset is a public image set that was collected as part of the research work described in [12]. The dataset contains 3,542 positive and 12,180 negative images for training and testing pedestrian detectors. Images are normalized and have a resolution of 64 × 128 pixels.

The mammography dataset contains lesions from a database of mammograms provided by local Hospitals. Lesions have been marked and extracted from the original images by radiologists. The dataset has 1,339 images which contain lesions and 4,300 images cropped from the background. All images have been scaled to have a resolution of 500 × 500 pixels.

### Feature sets

Although recent work based on compact bag-of-patterns (CBoPs) descriptors [32], hyperspectral hypergraph of SIFT features [33], or the sparse auto-encoder (SAE) feature learning method [34, 35] have shown good results recently, in this work features are kept simple in order to better compare detector structures. For all datasets, two feature sets were obtained: Haar-like [1] and statistical [4, 36] features.

The set of Haar-like features is traditionally used in Viola-Jones cascade detectors. Haar-like features are commonly used in object detection and are similar to Haar wavelets [37]. Viola and Jones proposed these features instead of original pixel values due to the complexity of pixel-based detectors [1]. Haar-like features consider adjacent rectangular regions and start by summing up pixel intensities in each region. Then, they calculate the difference between these sums and finally, these differences are used by the detector. The main advantage of Haar-like features over other features is their speed.

A simple rectangular Haar-like feature can be defined as the difference of the sum of the pixel values inside two adjacent rectangles. These rectangles can be in any position and scale within the original image. Basic Haar-like features are based on two adjacent rectangles. Viola and Jones also defined 3-rectangle and 4-rectangle features. Thus, each feature represents a specific characteristic of a particular area of the image, such as the existence (or not) of edges or texture changes.

**Fig 4:**
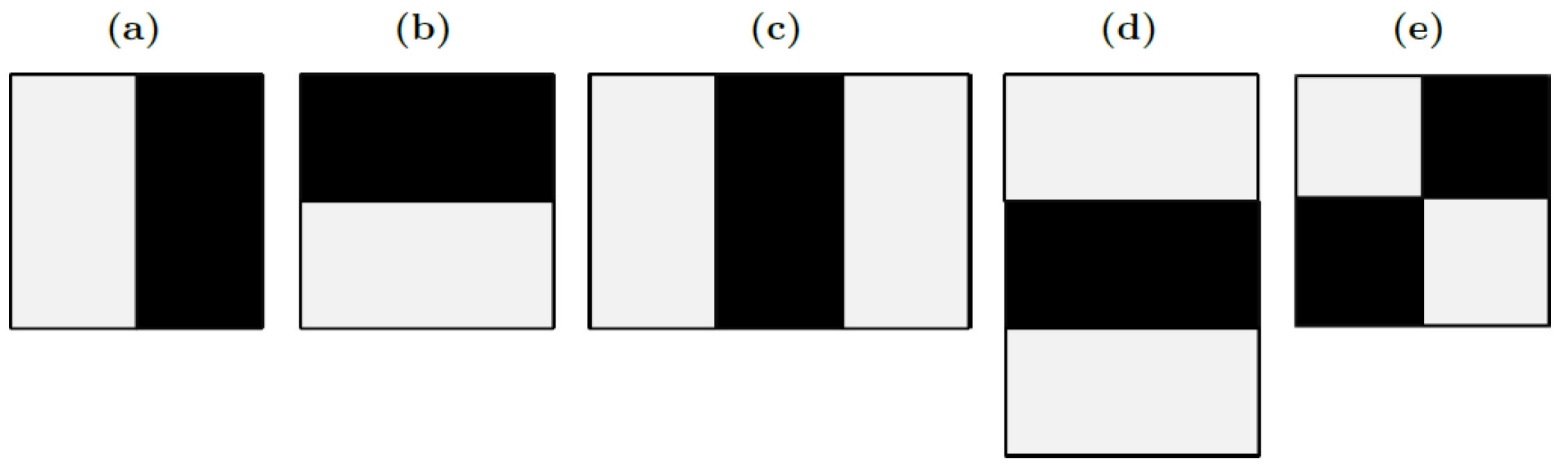
Base Haar-like rectangle features.

Fig 4 depicts the basic set of Haar-like features used by Viola and Jones [1]. This set consists of three types of features based on 2, 3 and 4 rectangles. In the case of the 2-rectangle features, the obtained value represents the difference between the sum of the pixels within two rectangular regions. These regions have the same size and shape and may be horizontally or vertically adjacent. Features based on three rectangles calculate the sum within the two outer rectangles and this value is subtracted from the sum in the central rectangle. Like 2-rectangle features, these rectangles may be horizontally or vertically adjacent. Finally, the 4-rectangle features calculate the difference between diagonal pairs of rectangles.

The statistical feature sets have been drawn from the histograms and the co-occurrence matrices by means of the 1^*st*^-order statistics (mean, mode, variance, 1^*st*^ quartile, 2^*nd*^ quartile, 3^*rd*^ quartile, interquartile range, value range, entropy, asymmetry, and kurtosis) and 2^*nd*^-order texture statistics (Haralick coefficients) [38]. These features are listed in detail in [36]. The angles and distances used to calculate the co-occurrence matrices are *α* = {0^*o*^, 45^*o*^, 90^*o*^, 135^*o*^} and *d* = {1, 3, 5, 10, 15} respectively. These features are shown with their definitions in Tables 2 and 3.

**Table 2:** First-order statistics.

Feature	Definition
1. Mean	$\sum_{i=0}^{N-1}ih(i)$
2. Mode	*i*∣*h*(*i*) = *max*(*h*)
3. Variance	$\sum_{n=0}^{N-1}{\left(i-\mu \right)}^{2}h(i)$
4. 1st quartile	$\frac{N}{4},even\_N$
	$\frac{N+1}{4},odd\_n$
5. 2nd quartile	$\frac{2N}{4},even\_N$
	$\frac{2N+1}{4},odd\_n$
6. 3rd quartile	$\frac{3N}{4},even\_N$
	$\frac{3N+1}{4},odd\_N$
7. Interquartile Range	#6—#4
8. Minimum	*i*∣*h*(*i*) > 0 and ∄*j* < *i*∣*h*(*j*) > 0
9. Maximum	*i*∣*h*(*i*) > 0 and ∄*j* > *i*∣*h*(*j*) > 0
10. Value Range	*Max*(*h*(*i*)) − *Min*(*h*(*i*))
11. Entropy	$\sum_{i=0}^{N-1}h(i)log(h(i))$
12. Asymmetry	$\frac{1}{\sigma^{3}}\sum_{n=0}^{N-1}{\left(i-\mu \right)}^{3}h(i)$
13. Kurtosis	$\frac{1}{\sigma^{4}}\sum_{n=0}^{N-1}{\left(i-\mu \right)}^{4}h(i)$
Where:	
*h* is the normalized image histogram	
*N* is the number of gray levels	

**Table 3:** Second-order statistics.

Feature	Definition
1. Energy	$\sum_{i=0}^{N-1}\sum_{j=0}^{N-1}p{\left(i,j\right)}^{2}$
2. Correlation	$\frac{\sum_{i=0}^{N-1}\sum_{j=0}^{N-1}(ij)p(i,j)-\mu_{x}\mu_{y}}{\sigma_{x}\sigma_{y}}$
3. Contrast	$\sum_{n=0}^{N-1}n^{2}\sum_{i=0}^{N-1}\sum_{j=0}^{N-1}p(i,j)$; ∣*i* − *j*∣ = *n*
4. Variance	$\sum_{i=0}^{N-1}\sum_{j=0}^{N-1}{\left(i-\mu \right)}^{2}p(i,j)$
5. Sum Average	$\sum_{i=0}^{2(N-1)}ip_{x+y}(i)$
6. Sum Entropy	$\sum_{i=0}^{2(N-1)}p_{x+y}(i)log(p_{x+y}(i,j))$
7. Sum Variance	$-\sum_{i=0}^{2(N-1)}{\left(i-\#6\right)}^{2}p_{x+y}(i)$
8. Entropy	$-\sum_{i=0}^{N-1}\sum_{j=0}^{N-1}p(i,j)log(p(i,j))$
9. Difference Variance	$\sum_{i=0}^{N-1}i^{2}p_{x-y}(i)$
10. Difference Entropy	$-\sum_{i=0}^{N-1}p_{x-y}(i)log(p_{x-y}(i,j))$
11. Correlation Inf. 1	$\frac{HXY-HXY1}{max(HX,HY)}$
12. Correlation Inf. 2	$\sqrt{1-exp(-2(HXY2-HXY)}$
13. Homogeneity 1	$\sum_{i=0}^{N-1}\sum_{j=0}^{N-1}\frac{p(i,j)}{1+{\left(i-j\right)}^{2}}$
14. Homogeneity 2	$\sum_{i=0}^{N-1}\sum_{j=0}^{N-1}\frac{p(i,j)}{1+|i-j|}$
15. Cluster Shade	$\sum_{i=0}^{N-1}\sum_{j=0}^{N-1}{\left(i+j-\mu_{x}-\mu_{y}\right)}^{3}p(i,j)$
16. Cluster Prominence	$\sum_{i=0}^{N-1}\sum_{j=0}^{N-1}{\left(i+j-\mu_{x}-\mu_{y}\right)}^{4}p(i,j)$
17. Autocorrelation	$\sum_{i=0}^{N-1}\sum_{j=0}^{N-1}(ij)p(i,j)$
18. Dissimilarity	$\sum_{i=0}^{N-1}\sum_{j=0}^{N-1}|i-j|p(i,j)$
19. Maximum Probability	*max*(*p*(*i*, *j*)); *i*, *j* = [0…*N* − 1]
Where:	
*p* is the image co-occurrence matrix	
*N* is the number of gray levels	
$\mu_{x}=\sum_{i=0}^{N-1}\sum_{j=0}^{N-1}ip(i,j)$; $p_{x}(i)=\sum_{j=0}^{N-1}p(i,j)$	
$\mu_{y}=\sum_{i=0}^{N-1}\sum_{j=0}^{N-1}jp(i,j)$; $p_{y}(j)=\sum_{i=0}^{N-1}p(i,j)$	
$\sigma_{x}=\sqrt{\sum_{i=0}^{N-1}p_{x}(i){\left(i-\mu_{x}\right)}^{2}}$; $\sigma_{y}=\sqrt{\sum_{j=0}^{N-1}p_{y}(i){\left(i-\mu_{y}\right)}^{2}}$	
$p_{x+y}(k)=\sum_{i=0}^{N-1}\sum_{j=0}^{N-1}p(i,j)$, *i*+*j* = *k*, *k* = 0…2(*N* − 1)	
$p_{x-y}(k)=\sum_{i=0}^{N-1}\sum_{j=0}^{N-1}p(i,j)$, ∣*i* − *j*∣ = *k*, *k* = 0…*N* − 1	
$HXY=-\sum_{i=0}^{N-1}\sum_{j=0}^{N-1}p(i,j)log(p(i,j))$	
$HXY1=-\sum_{i=0}^{N-1}\sum_{j=0}^{N-1}p(i,j)log(p_{x}(i)p_{y}(j))$	
$HXY2=-\sum_{i=0}^{N-1}\sum_{j=0}^{N-1}p_{x}(i)p_{y}(j)log(p_{x}(i)p_{y}(j))$	
*HX* and *HY* are the entropies of *p*_*x*_ and *p*_*y*_	

## Results and Discussion

In this section, the cascades proposed by Viola and Jones [1], Chen and Chen [13], the Soft cascade [14] and the proposed cascade with sample selection (Algorithm 1 in Table 1) are compared. All of these algorithms have been modified and implemented to have the same boosting algorithm, AdaBoost [15], and to use Decision Stumps as weak learners [39]. Other ensemble classification methods can be used as stage classifiers [40]. In addition, two sets of features (Haar-like and statistical) were obtained for the three databases (CBCL, INRIA, and Mammography) and used to train all the detectors. In the experiments, the performance of the different cascade detectors was assessed by means of the (*10fcv*) method for training and testing. The *kfcv* training/testing process was carried out fixing the same training parameters for all cascade detectors. The minimum stage Dr was set to 1 and the maximum stage FPr was set to 0.5. The global FPr was adjusted to values 0.02, 0.05, 0.1, and 0.2. Therefore, comparisons are to be made only over the cascade structures and they do not include any variation on the boosting technique, the weak learner, or the features used.

On the other hand, since the proposed method selects a number of misclassified negative samples (see Section Proposed method), the other cascades were modified to randomly select the same number of samples. This was done in order to ensure that all methods access the same amount of samples. The same random selection was performed in the first stage of the proposed cascade framework due to the lack of previous stage confidence information.

**Fig 5:**
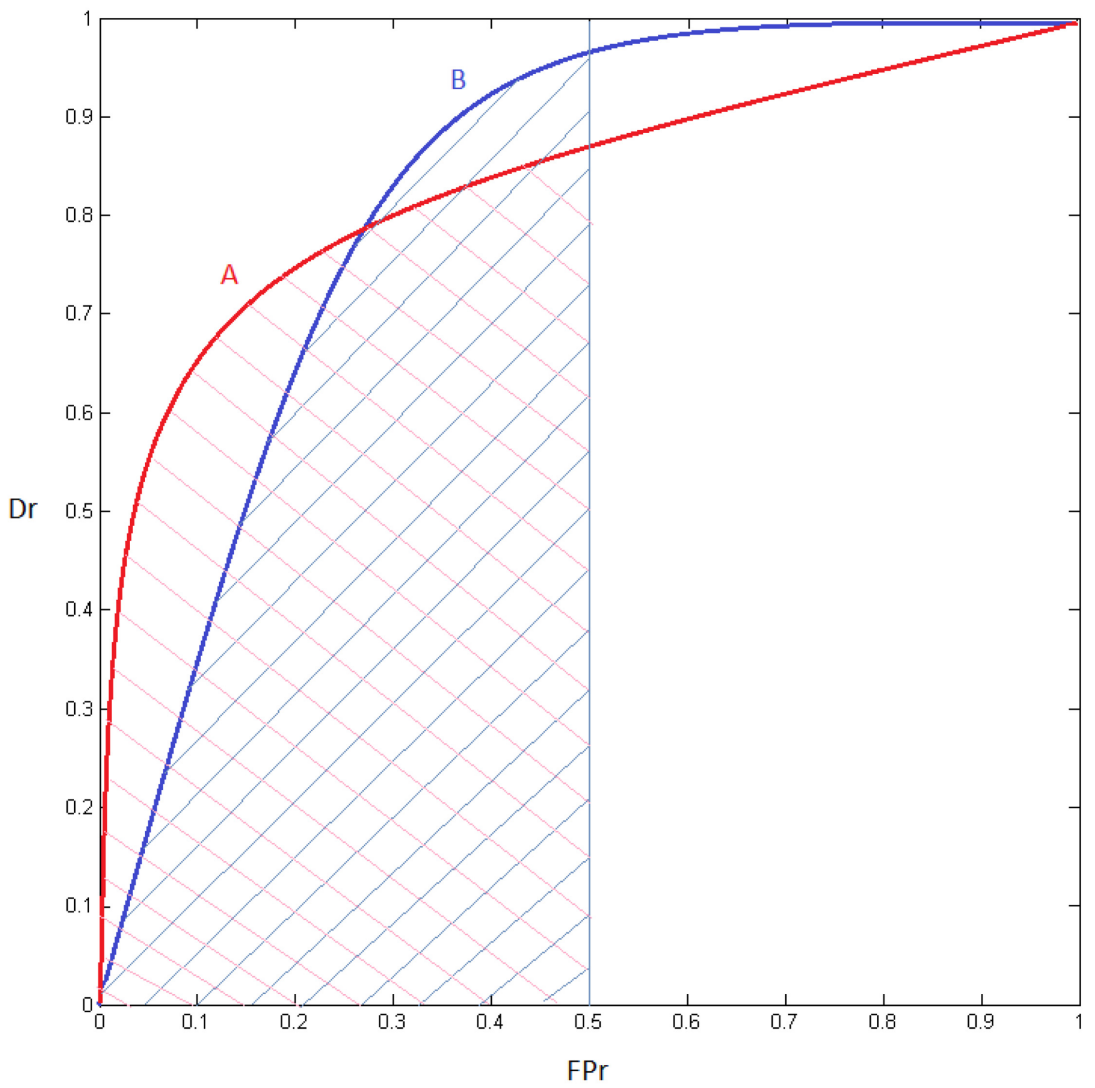
Two ROCs, A and B, with the same AUC but different pAUC.

**Fig 6:**
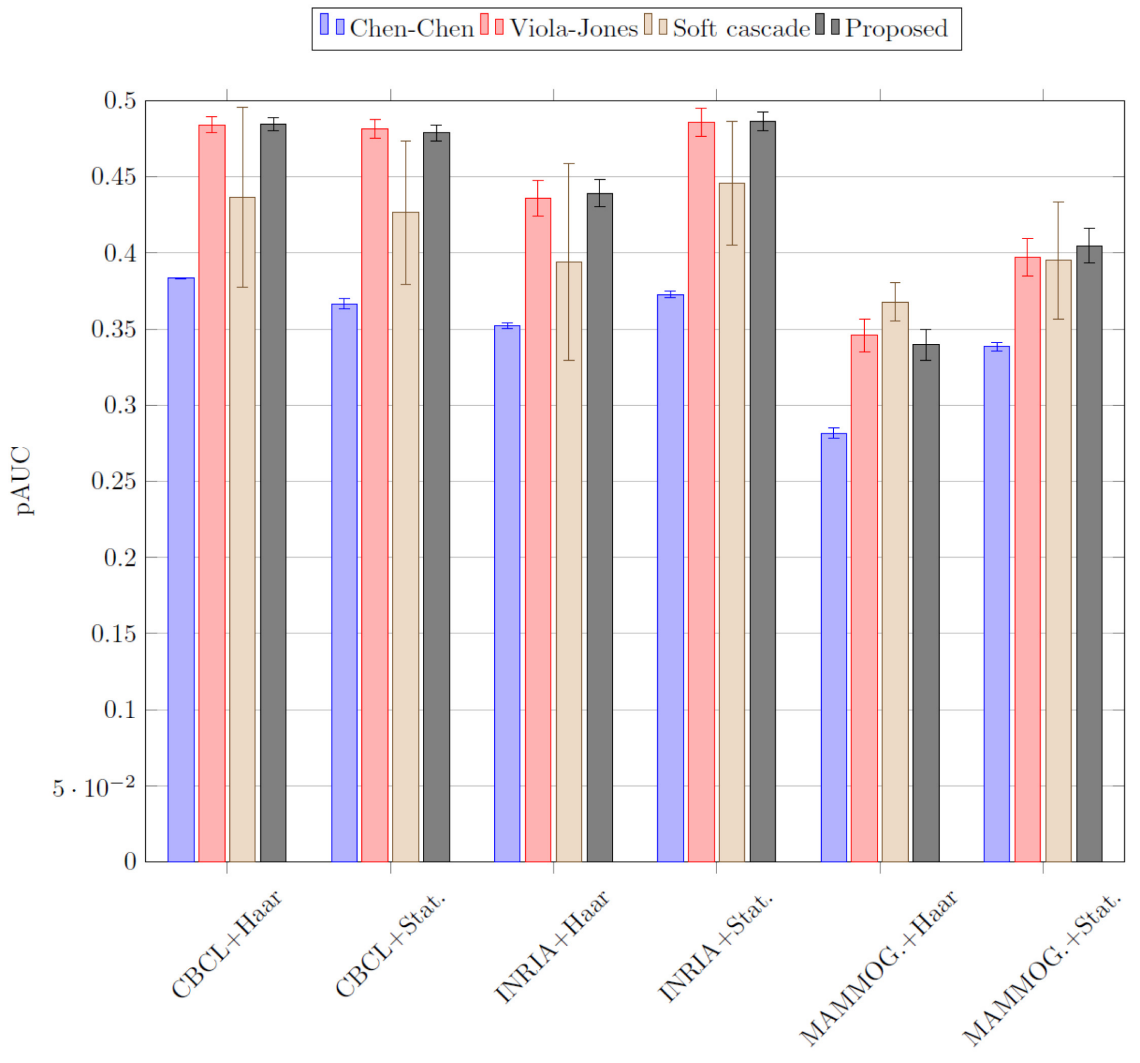
Average pAUC results of the different cascade detectors using the six dataset and feature set combinations. The lines on top of the bars represent the standard deviation.

In order to better examine the results and select the best detector independently of the class distribution over the population, Receiver Operating Characteristic curves (ROCs) were obtained [41]. Once ROC curves are obtained for each detector and global FPr target value, the partial area under the curve (pAUC) is calculated [42–44]. This has been proposed in the literature as an alternative measure to the full AUC [43]. The partial AUC summarizes test accuracy over a relevant region of the ROC curve. To see the importance of this, consider the two ROC curves of Fig 5. If the two curves are compared, it is possible to notice that curve *A* represents a better performance than curve *B* on the first part, that is, when FPr is low. Thus, the higher pAUC value on the first part of the ROC curve, the better Dr over the selected FPr interval. In this work, the FPr interval selected to compute the pAUC ranges between 0 and 0.5 since 0.5 is the random guessing error of a two-class problem. Table 4 shows the average pAUCs obtained for each detector, database and feature set. A graphical representation of the results is shown in Fig 6.

**Table 4:** pAUC results.

	CBCL		INRIA		Mammography	
	Haar-like	Statistical	Haar-like	Statistical	Haar-like	Statistical
*(a) Chen-Chen cascade detector*						
pAUC	0.3833	0.3666	0.3521	0.3727	0.2816	0.3384
*σ*	0.000	0.0032	0.0020	0.0023	0.0032	0.0027
*(b) Viola-Jones cascade detector*						
pAUC	0.4842	**0.4814**	0.4359	0.4860	0.3458	0.3972
*σ*	0.0051	0.0061	0.0116	0.0093	0.0109	0.0123
*(c) Soft cascade detector*						
pAUC	0.4364	0.4264	0.3940	0.4458	**0.3679**	0.3950
*σ*	0.0591	0.0468	0.0648	0.0406	0.0125	0.0386
*(d) Proposed cascade detector with FP selection*						
pAUC	**0.4845**	0.4788	**0.4391**	**0.4865**	0.3398	**0.4047**
*σ*	0.0044	0.0054	0.0089	0.0063	0.0102	0.0113

Results obtained from applying the different cascade detectors over the different dataset and feature set combinations. The table shows the average pAUC values with their corresponding standard deviation *σ*. The best pAUC for each pair of database and feature set is highlighted in bold.

Since pAUC differences can be small in some cases, the standard deviation, *σ*, has also been computed for each case in order to compare the results obtained. From the results in Table 4, it is possible to conclude that the proposed cascade detector with sample selection is better than the other methods considered. It obtains better pAUC than the rest of the detectors with random sample selection for CBCL+Haar-like, INRIA+Haar-like, INRIA+Statistical and Mammography+Statistical. For the CBCL+Statistical combination, the detector which obtained higher pAUC was the Viola-Jones detector with random sample selection. For Mammography+Haar-like the best results have been obtained with the Soft cascade. In these two specific cases, a lower *σ* has been achieved by the proposed cascade. The smallest values of *σ* are obtained by the Chen-Chen detector but it also has the lowest pAUC values. These values range between 0 and 0.0032. The following detector with the smallest *σ* values is the proposed one with values ranging between 0.0044 and 0.0113. Values of *σ* between 0.0051 and 0.0123 are obtained by the Viola-Jones detector. Finally, the Soft cascade obtains the highest *σ* values ranging between 0.0125 and 0.0648.

## Conclusions

In this work a cascade detector with sample selection is proposed for improving cascade detectors. The method is based on adding new stages with a selection of the accumulated misclassified negative samples generated from running the detector until the previous stage, keeping the same number of positive and negative samples.

The proposed sample selection was compared with other cascade detectors using random sample selection in six different scenarios combining three different datasets with two different feature sets. The effectiveness of the methods was assessed through the average partial AUC from the ROC curves obtained with 10*fcv*. The results show that the proposed cascade detector with sample selection obtains better pAUC and smaller *σ* than the rest of the detectors in all cases except for CBCL+Haar-like and Mammography+Statistical database and feature set combination. However, in these two cases the obtained values from *σ* are smaller for the proposed method. Moreover, the Soft cascade method shows large variations between the results obtained in most of the cases, which is not appropriate while training detectors.

Since the proposed approach does not rely on a specific type of cascade classifiers, it can be generalised to other cascade types. In future work, the sample selection and other training parameters for training cascades will be analysed jointly. We plan to employ our method for medical lesion detection problems which require examining a large number of negative regions.
